# Reduced hippocampal volumes and memory deficits in adolescents with single ventricle heart disease

**DOI:** 10.1002/brb3.1977

**Published:** 2021-01-07

**Authors:** Nancy A. Pike, Bhaswati Roy, Stefanie Moye, Cristina Cabrera‐Mino, Mary A. Woo, Nancy J. Halnon, Alan B. Lewis, Rajesh Kumar

**Affiliations:** ^1^ UCLA School of Nursing University of California Los Angeles Los Angeles CA USA; ^2^ Division of Pediatric Cardiology University of California Los Angeles Los Angeles CA USA; ^3^ Division of Pediatric Cardiology Children's Hospital Los Angeles Los Angeles CA USA; ^4^ Departments of Anesthesiology University of California Los Angeles Los Angeles CA USA; ^5^ Radiological Sciences University of California Los Angeles Los Angeles CA USA; ^6^ Bioengineering University of California Los Angeles Los Angeles CA USA; ^7^ Brain Research Institute University of California Los Angeles Los Angeles CA USA

**Keywords:** cognition, congenital heart disease, gray matter, magnetic resonance imaging

## Abstract

**Introduction:**

Adolescents with single ventricle congenital heart disease (SVHD) show functional deficits, particularly in memory and mood regulation. Hippocampi are key brain structures that regulate mood and memory; however, their tissue integrity in SVHD is unclear. Our study aim is to evaluate hippocampal volumes and their associations with memory, anxiety, and mood scores in adolescents with SVHD compared to healthy controls.

**Methods:**

We collected brain magnetic resonance imaging data from 25 SVHD (age 15.9 ± 1.2 years; 15 male) and 38 controls (16.0 ± 1.1 years; 19 male) and assessed memory (Wide Range Assessment of Memory and Learning 2, WRAML2), anxiety (Beck Anxiety Inventory, BAI), and mood (Patient Health Questionnaire 9, PHQ‐9) functions. Both left and right hippocampi were outlined and global volumes, as well as three‐dimensional surfaces were compared between groups using ANCOVA and associations with cognitive and behavioral scores with partial correlations (covariates: age and total brain volume).

**Results:**

The SVHD group showed significantly higher BAI (*p* = .001) and PHQ‐9 (*p* < .001) scores, indicating anxiety and depression symptoms and significantly reduced WRAML2 scores (*p* < .001), suggesting memory deficits compared with controls. SVHD group had significantly reduced right global hippocampal volumes (*p* = .036) compared with controls, but not the left (*p* = .114). Right hippocampal volume reductions were localized in the CA1, CA4, subiculum, and dentate gyrus. Positive correlations emerged between WRAML2 scores and left (*r* = 0.32, *p* = .01) and right (*r* = 0.28, *p* = .03) hippocampal volumes, but BAI and PHQ‐9 did not show significant correlations.

**Conclusion:**

Adolescents with SVHD show reduced hippocampal volumes, localized in several sites (CA1, CA4, subiculum, and dentate gyrus), which are associated with memory deficits. The findings indicate the need to explore ways to improve memory to optimize academic achievement and ability for self‐care in the condition.

## Graphical abstract

Right hippocampal volume was significantly reduced in adolescents with SVHD compared to controls, localized in several sites (CA1, CA4, subiculum, and dentate gyrus), which are associated with memory deficits and trended toward significance in anxiety and mood.

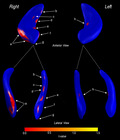

## 1 INTRODUCTION

Single ventricle heart disease (SVHD), which is a subgroup of critical congenital heart disease (CHD) (Oster et al., 2013), requires a series of staged palliative surgical procedures at a young age with the culmination of the Fontan operation leaving the patient with one ventricle that pumps blood in series to the pulmonary and systemic circulations. Advances in diagnosis, surgical techniques, and medical management have resulted in greater life expectancies for SVHD children and now expected to survive into adulthood (Hoffman & Kaplan, 2002). Up to 50% of SVHD patients suffer from memory, anxiety, and mood impairments that can negatively affect behavior, academic performance, employability, self‐care ability, and overall quality of life (Pike et al., 2016). Although the underlying etiology for these impairments is not completely understood, delayed brain maturation and nonspecific brain structural injury have been reported at birth and pre‐ and postoperative cardiac surgery (Miller et al., 2007; Pike et al., 2018). However, the status of brain structures that control memory, anxiety, and mood functions in SVHD is unclear.

Although multiple brain sites, including the anterior thalamus, fornix fibers, mammillary bodies, amygdala, insula, and anterior cingulate are involved in cognition and mood control (Shohamy & Turk‐Browne, 2013). The hippocampal sites are key areas for such regulation (Drevets et al., 2008), which are particularly prone to injury during development and are highly vulnerable to hypoxic and ischemic conditions (Cooper et al., 2015). While the hippocampus status after palliative surgery is uncertain in SVHD, hippocampal volumes are significantly decreased in non‐CHD patients at risk for memory, anxiety, and mood impairment (i.e., premature infants [Peterson et al., 2000], epilepsy [Shamim et al., 2009], and infant stroke [Gold & Trauner, 2014]).

Recent studies showed hippocampal volume correlations with memory impairments (i.e., working memory, episodic memory, and verbal comprehension) in a group of patients with transposition of the great arteries who underwent the arterial switch operation (Munoz‐Lopez et al., 2017) and subjects with multiple types of CHD after surgical intervention (Fontes et al., 2019; Latal et al., 2016). However, these studies utilized different CHD populations, included subjects with other potential risk factors for hippocampal volume loss (i.e., prematurity, previous extracorporeal membrane oxygenation (ECMO) use, and stroke), different cognitive measures, and methods to assess hippocampal volumes (i.e., gold standard manual tracings vs. automated processes) (Fontes et al., 2019; Latal et al., 2016; Munoz‐Lopez et al., 2017). Furthermore, there are no published studies that have examined the correlations between memory, anxiety, and mood scores to hippocampal volumes in the SVHD population using objective measures (i.e., manual tracing of structures).

Our study aims were to evaluate hippocampal volumes and 3D surfaces, using magnetic resonance imaging (MRI)‐based high‐resolution T1‐weighted imaging, in adolescents with SVHD compared with controls and their potential associations with memory, anxiety, and mood deficits.

## 2 METHODS

### 2.1 Study design and participants

Using comparative and correlational study designs, 63 adolescents (25 SVHD [mean age, 15.9 ± 1.2 years; 15 male] and 38 controls [mean age, 16.0 ± 1.1 years; 19 male]) were enrolled. Adolescents (ages 14–18 years) with SVHD, who have undergone Fontan completion, were recruited via research flyers or provider referrals from the University of California Los Angeles (UCLA) and Children's Hospital of Los Angeles (CHLA) in Southern California. Healthy controls were recruited from local high schools, surrounding community, and word of mouth. All controls were screened and excluded for chronic medical or psychiatric conditions, and previous head trauma. Exclusion criteria for SVHD and controls were claustrophobia, nonremovable metal (such as braces, pacemakers), severe developmental delay (e.g., cerebral palsy or severe hypoxic‐injury) precluding active study participation, diagnosis of depression, premature birth (<37 weeks gestation), history of ECMO use, or previous documented stroke and cardiac arrest. Controls were matched to an SVHD subject for age (±1 year), sex, and ethnicity. Clinical and demographic information was collected from participants and their medical records for the SVHD group.

Parental permission and assent were obtained from all participants under 18 years of age, and written informed consent was obtained from participants over 18 years before data collection. The Institutional Review Boards at UCLA and CHLA approved the study protocol.

### 2.2 Cognition assessment

Multiple aspects of cognition, especially memory was assessed using the Wide Range Assessment of Memory and Learning, Version 2 (WRAML2). The WRAML2 is an administered assessment of memory and learning which includes verbal and visual memory, attention/concentration, working memory, and visual and verbal memory recognition (Pike et al., 2016). These six subtests make up the WRAML2 and subscores from each section are summed to yield the general memory index (GMI) and general recognition index (GRI) scores (mean score = 100, *SD* ± 15, with a score ≤ 85 considered impaired) (Sheslow & Wayne, 2003). The alpha reliabilities for the core subtests range from 0.85 to 0.94 (Sheslow & Wayne, 2003).

Short‐term memory was also assessed by administration of the 5‐word recall (delayed memory recall) subscale of the Montreal Cognitive Assessment (MoCA). This test is often used as a cognitive screener and measures a multitude of intellectual aspects, such as visual‐spatial skills, executive function, delayed memory recall, attention, concentration, naming, and language (Nasreddine et al., 2005). Scores range from 0 to 30 (<26 is abnormal). The MoCA test has been validated in the adolescent CHD and general population with a Cronbach's alpha of 0.8 (Pike et al., 2017).

### 2.3 Anxiety and depression assessment

Anxiety was measured by self‐reported questionnaire using the Beck Anxiety Inventory (BAI) (Beck et al., 1988), which consists of 21‐items each rated on a scale from 0 to 3 with a total score ranging from 0 to 63. A higher score (>36) indicates a clinically significant level of anxiety. The BAI has been validated for use in adolescents (Osman et al., 2002) and in previous CHD studies with a Cronbach's alpha of 0.93 (Beck et al., 1988; Pike et al., 2016).

Depression was measured by self‐reported questionnaire using the Patient Health Questionnaire‐9 (PHQ‐9), which consists of 9 items that measure symptoms of depression. The scores range from 0 to 27: scores (5–9) identify minimal symptoms, (10–14) moderate, (15–19) moderate‐severe, and (20≥) severe depression (Kroenke et al., 2001). The PHQ‐9 has been previously used in adolescents (Richardson et al., 2010) and in the CHD population (Pike et al., 2016) with a Cronbach's alpha range of 0.86–0.89 (Kroenke et al., 2001).

### 2.4 Socioeconomic status

Socioeconomic status (SES) reflects the annual household income derived from each subject's residential postal zip code. Income for each participant was calculated from the American Community Survey data available on Population Studies Center, Institute for Social Research (https://www.census.gov/programs‐surveys/acs/).

### 2.5 Magnetic resonance imaging

While participants lay supine, brain‐imaging data were acquired using a 3.0‐Tesla MRI scanner (Siemens, Magnetom Tim‐Trio and Prisma, Erlangen, Germany). Foam pads were placed on either side of the head to minimize head movement. The magnetization prepared rapid acquisition gradient‐echo (MPRAGE) sequence [repetition time (TR) = 2200 ms; echo time (TE) = 2.34/2.41 ms; inversion time = 900 ms; flip angle (FA) = 9°; matrix size 320 × 320; field of view (FOV) = 230 × 230 mm; slice thickness = 0.9 mm) was used to acquire two separate high‐resolution T1‐weighted image series. Whole‐brain proton density (PD) and T2‐weighted images (TR = 10,000 ms; TE1, TE2 = 12, 123/124 ms; FA = 130°) were collected with a dual‐echo turbo spin‐echo pulse sequence in the axial plane (230 × 230 mm FOV, 256 × 256 matrix size, 3.5 mm slice thickness, and no interslice gap). T1‐, T2‐, and PD‐weighted images were visually evaluated to ensure the absence of movement artifacts or gross brain pathology, such as infarcts or mass lesions. If head motion occurred during MRI data acquisition, scans were repeated. In addition, all MRIs were evaluated by a neuroradiologist who was blinded to group assignment.

### 2.6 Image processing

The preprocessing of the images was performed using SPM12 software (Wellcome Department of Cognitive Neurology, UK; http://www.fil.ion.ucl.ac.uk/spm/) and MATLAB‐ based (The MathWorks Inc) custom software. Both high‐resolution T1‐weighted volumes of each subject were realigned and averaged to improve signal‐to‐noise ratios. The averaged images were bias‐corrected for any potential image signal intensity variations using the unified segmentation approach, and reoriented into a common Montreal Neurological Institute (MNI) space, and resampled (voxel size, 0.7 × 0.7 × 0.7 mm). The reoriented images were used for manual outlining of hippocampal structures.

The bias‐corrected and reoriented images of each individual subject were partitioned into gray, white, and cerebrospinal fluid (CSF) probability maps using the unified segmentation method. The automatic segmentation method is based on tissue classification approach, where voxels are assigned to a tissue class based on their intensities. The intensity values are used to generate probability maps based on probability of belonging to each class (Ashburner & Friston, 2005). All voxels with a probability value > 0.5 for each gray, white, and CSF probability maps were counted, and whole‐brain gray, white, and CSF volumes were calculated. Whole‐brain gray and white matter volumes were added to determine total brain volume (TBV).

### 2.7 Hippocampal tracing and volume quantification

Manual ROI measures of the left and right hippocampi were performed on the reoriented and resampled T1‐weighted images in each subject using the MRIcron software (McCausland Center for Brain Imaging, University of South Carolina, Columbia, South Carolina). One investigator, blinded to subject diagnosis, performed left and right hippocampal tracings in all subjects. The hippocampal sites were outlined initially in the sagittal view, and then coronal and axial views were utilized as confirmation of tracing accuracy. The hippocampus main body (CA1–CA4), dentate gyrus, and subiculum were traced in each image. The head of the hippocampus was traced from inferior horn of the lateral ventricle separating from the amygdala. The inferior margin of the hippocampus was outlined to include the subicular complex and the uncal cleft with the border separating the subicular complex from the parahippocampal gyrus. For the body of the hippocampus, the delineation included the subicular complex, hippocampus proper, dentate gyrus, alveus, and fimbria and excluded the cortex of the parahippocampal gyrus. Hippocampal tail included the subicular complex, hippocampus proper, dentate gyrus, alveus, and fimbria excluding the crus of the fornix, isthmus of the cingulate gyrus, and parahippocampal gyrus (Watson et al., 1992). The total hippocampal volumes were then calculated by summing all voxels and multiplying by a voxel volume.

### 2.8 Intra‐ and intertracing reliability analysis

We examined intratracer reliability for outlining hippocampus structures by re‐outlining in 9 randomly‐selected SVHD and 7 control subjects by the same investigator who outlined structures in all SVHD and control subjects. A second investigator retraced hippocampus in same 16 subjects (9 SVHD and 7 control), from which inter‐rater reliabilities were calculated for the two investigators. Inter‐ and intratracing reliabilities were calculated using intraclass correlation (ICC).

### 2.9 Statistical analysis

Demographic data were compared with independent samples *t* tests (two‐ tailed) for normally distributed data, Mann–Whitney *U* test for non‐normally distributed data, and with the chi‐square test for categorical variables. The normality of each variable for SVHD and control subjects was evaluated using Shapiro–Wilk tests. ANCOVA was used to compare hippocampal volumes between SVHD and control groups with covariates of age and TBV. Memory, anxiety, and mood scores were compared between SVHD and control groups using independent samples *t* tests or Mann–Whitney *U* tests based on their normality. Partial correlations were used to identify relationships between hippocampal volumes, memory, anxiety, and mood scores (covariates, age, and TBV) in SVHD subjects. A *p* < .05 was considered as statistically significant.

### 2.10 3D Surface morphometry

We used SPHARM‐MAT, a Fourier transform technique to visualize regional hippocampal volume changes between SVHD and control subjects. Three‐dimensional **(**3D) mesh files were created from individual hippocampal tracings, and the topology of voxel surfaces was fixed to sphere. Spherical parametrization, expansion, and surface alignment was performed on all the 3D mesh files to perform group analyses (ANCOVA; covariates, age, and TBV). The subregion hippocampal volume loss in SVHD compare with control subjects was overlaid onto averaged 3D hippocampus surface models, determined by averaging all SVHD and control surface models.

## 3 RESULTS

### 3.1 Demographic and clinical characteristics

Demographic, memory, anxiety, and mood scores of SVHD and control groups are shown in Table 1. No significant differences in age, sex, ethnicity, or handedness appeared between SVHD and control groups. However, SES was significantly lower in the SVHD group compared with controls. The total MoCA and all subscales, except orientation, were significantly lower in SVHD than controls (*p* < .05). In addition, WRAML2 GMI and GRI scores were significantly reduced and the other subdivisions, including verbal and visual memory, attention/concentration, working memory, and visual and verbal recognition, in SVHD compared with controls (*p* < .01). The BAI and PHQ‐9 scores were significantly higher in SVHD over controls (*p* < .005). TBV was significantly smaller in the SVHD group compared to controls (1.14 ± 0.12 versus 1.23 ± 0.12 L; *p* = .005), after correcting for age.

**Table 1:** Demographic, Memory, Mood, and Anxiety Characteristics of SVHD and Controls

Variables	SVHD *n* = 25	Control *n* = 38	*p*, *t*/*χ* ^2^/*U* Values	*df*	95% Confidence interval
Mean ± *SD*/ *n* (%)					
Age, median (IQR)	16.0 (15.0–17.0)	16.0 (15.0–17.0)	0.67, 446	–	0.66–0.68
Gender Male (%)	15 (60%)	19 (50%)	0.44, 0.61	1	0.2–1.9
Ethnicity (%) White Hispanic Other	13 (52%) 10 (40%) 2 (8%)	20 (50%) 16 (42%) 2 (8%)	0.91, 0.20	2	–
Socioeconomic Status, median (IQR) (Annual Household Income)	$73,167.5 ($54068–$96,758) (*n* = 24)	$85,696.0 ($74,317.5–$116,633.0) (*n* = 37)	0.02, 287	–	0.018–0.023
Handedness Right (%)	23 (92%)	34 (92%)	0.74, 0.11	1	0.13–4.37
BMI (kg/m^2^), median (IQR)	21.1 (19.7–3.0)	21.9 (19.6–24.9)	0.45, 409.5	–	0.44–0.46
Total Brain Tissue Volume (L)	1.14 ± 0.14	1.23 ± 0.11	0.005, 2.9	61	0.03–0.16
MoCA Total, median (IQR)	23 (20.5–25.0)	29 (27.8–30.0)	<0.001, 38.5	–	0–0.0003
Visuospatial/ EF (MoCA), median (IQR)	4.0 (3.0–5.0)	5.0 (5.0–5.0)	<0.001, 187	–	0–0.0003
Naming (MoCA), median (IQR)	3.0 (3.0–3.0)	3.0 (3.0–3.0)	0.03, 418	–	0.05–0.06
Attention (MoCA), median (IQR)	4.0 (3.0–5.0)	6.0 (6.0–6.0)	<0.001, 175	–	0–0.0003
Language (MoCA), median (IQR)	2.0 (1.0–2.0)	3.0 (2.0–3.0)	<0.001, 196	–	0–0.0003
Abstraction (MoCA), median (IQR)	1.0 (1.0–2.0)	2.0 (2.0–2.0)	<0.001, 264	–	0–0.0003
Delayed Memory Recall (MoCA), median (IQR)	2.0 (0.5–3.0)	4.0 (4.0–5.0)	<0.001, 90	–	0–0.0003
Orientation (MoCA), median (IQR)	6.0 (6.0–6.0)	6.0 (6.0–6.0)	0.49, 446.5	–	0.54–0.56
WRAML2 (GMI) Total, median (IQR)	83.0 (79.0–92.5)	111 (104.8–118)	<0.001, 52	–	0–0.0003
Verbal Memory Index (GMI), median (IQR)	88.0 (82.0–95.5)	111 (99.3–114)	<0.001, 129.5	–	0–0.0003
Visual Memory Index (GMI)	98.4 ± 12.8	107.5 ± 10.2	0.003, 3.1	61	3.2–14.9
Attention/Concentration (GMI)	83.5 ± 11.4	109.6 ± 10.0	<0.001, 9.6	61	20.7–31.6
WRAML2 (GRI) Total	93.6 ± 12.1	112.1 ± 10.7	<0.001, 6.3	60	12.6–24.3
Working Memory Index (GRI), median (IQR)	90.0 (79.5–97.5)	113.5 (102.0–122.0)	<0.001, 70.5	–	0–0.0003
Verbal Recognition Index (GRI), median (IQR)	93.0 (86.5–104.0)	108.0 (96.0–115.0)	0.001, 196.5	–	0–0.0003
Visual Recognition Index (GRI), median (IQR)	90.5 (84.3–108.3)	109 (102.3–115.8)	<0.001, 191	–	0–0.0003
Beck Anxiety Inventory (BAI), median (IQR)	16.0 (8.0–25.5)	8.0 (3.8–11.0)	0.001, 251.5	–	0.001–0.002
Patient Health Questionnaire 9, median (IQR) (PHQ‐9)	6.0 (4.0–10.0)	3.5 (2.0–5.0)	<0.001, 235.5	–	0–0.0005

Abbreviations: BMI, body mass index; *df*, degrees of freedom; EF, Executive Function; GMI, General Memory Index; GRI, General Memory Recognition Index; IQR, interquartile range; MoCA, Montreal Cognitive Assessment; WRAML2, Wide Range Assessment of Memory and Learning Version 2.

^*^ *p* < .05.

Clinical characteristics of the SVHD cohort are listed in Table 2. The majority of SVHD subjects were a single right ventricle (67%), had an extracardiac Fontan (78%), only a small number with fenestration (26%) and residual cyanosis (26%), with O_2_ saturations less than 93%.

**Table 2:** Clinical characteristics of the SVHD group (*n* = 25)

Clinical Variables	*n* (%)
Single Ventricle Diagnosis:	
Hypoplastic Left Heart Syndrome	6 (24%)
DORV, Unbalanced AVC	6 (24%)
Tricuspid Atresia	4 (16%)
PA/ Unbalanced AVC	4 (16%)
PA/ IVS/ HRV	3 (12%)
Double Inlet Left Ventricle	2 (8%)
Ventricle Type (Right)	16 (67%)
Fontan Type (Extracardiac)	21 (78%)
Fontan Fenestration (Yes)	7 (26%)
Residual Cyanosis*	7 (26%)
Number of Surgeries ‐ mean (range)	3 (2–4)
Number of Medications – mean (range)	3 (1–5)

Abbreviations: AVC, Atrioventricular Canal; DORV, Double Outlet Right Ventricle; HRV, Hypoplastic Right Ventricle; IVS, Intact Ventricular Septum; PA, Pulmonary Atresia; SVHD, Single Ventricle Heart Disease.

* Oxygen saturation level < 93.

### 3.2 Tracing reliability

Intratracer reliability was high for hippocampus tracings (ICC = 0.95, *p* < .001, 95% confidence interval), indicating consistent tracings across all subjects. Inter‐rater reliability for hippocampi was in agreement between the two investigators (ICC = 0.82, *p* < .001, 95% confidence interval).

### 3.3 Global and localized hippocampal volume changes

**Figure 1:**
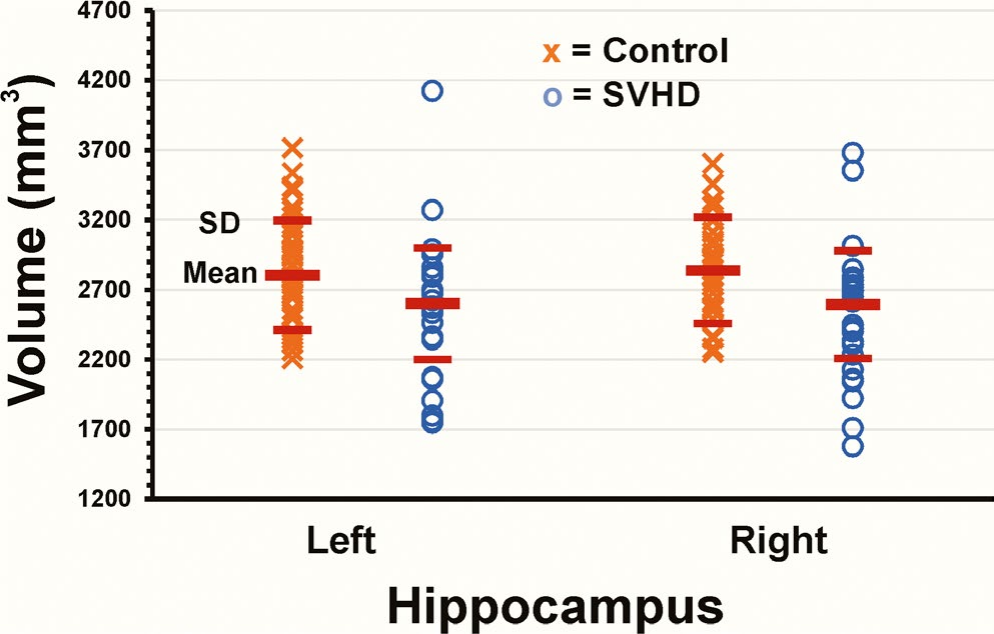
Scatter plots show the mean left and right hippocampal volumes (red horizontal bar) and standard deviations (red horizontal bars). The left and right hippocampal volumes are smaller in SVHD (O) versus Controls (X) (left, 2,627.1 mm^3^ versus 2,785.8 mm^3^; Right 2,616.7 mm^3^ versus 2,821.9 mm^3^), respectively

**Figure 2:**
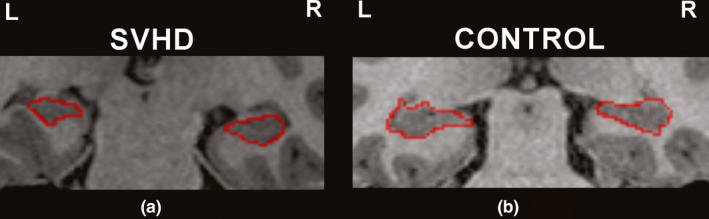
Coronal view of left and right hippocampi in a 15‐year‐old girl (a) with SVHD (left, 2,794.1 mm^3^; right 3,013.9 mm^3^) and an age‐ and sex‐matched control (b) (left, 3,269.1 mm^3^; right, 3,296.2 mm^3^)

SVHD subjects showed significantly smaller left and right hippocampal volumes in comparison with controls (Figure 1). However, no significant differences in left hippocampal volumes emerged between groups after correcting for age and TBV (Table 3). A large effect size was identified in both the left and right hippocampus (0.51 vs. 0.64), respectively. Figure 2 shows a coronal view of the left and right hippocampi in a 15‐year‐old girl (A) with SVHD and an age‐ and sex‐matched control (B).

**Table 3:** Hippocampal volumes of the SVHD and control groups

Hippocampal volumes	SVHD Mean ± *SD* (95% CI)	Controls Mean ± *SD* (95% CI)	*p, F* Value	*df*
Left (mm^3^)	2,627.1 ± 374.5 (2,477.2, 2,777)	2,785.8 ± 369.5 (2,665.8, 2,905.7)	0.114, 2.57	62
Right (mm^3^)	2,616.7 ± 362.1 (2,471.8, 2,761.6)	2,821.9 ± 357.2 (2,706, 2,937.9)	0.036, 4.61	62

Abbreviations: CI, confidence interval; *df*, degrees of freedom for adjusted total; SVHD, Single Ventricle Heart Disease. Corrected for age, and total brain volume.

* *p* < .05

**Figure 3:**
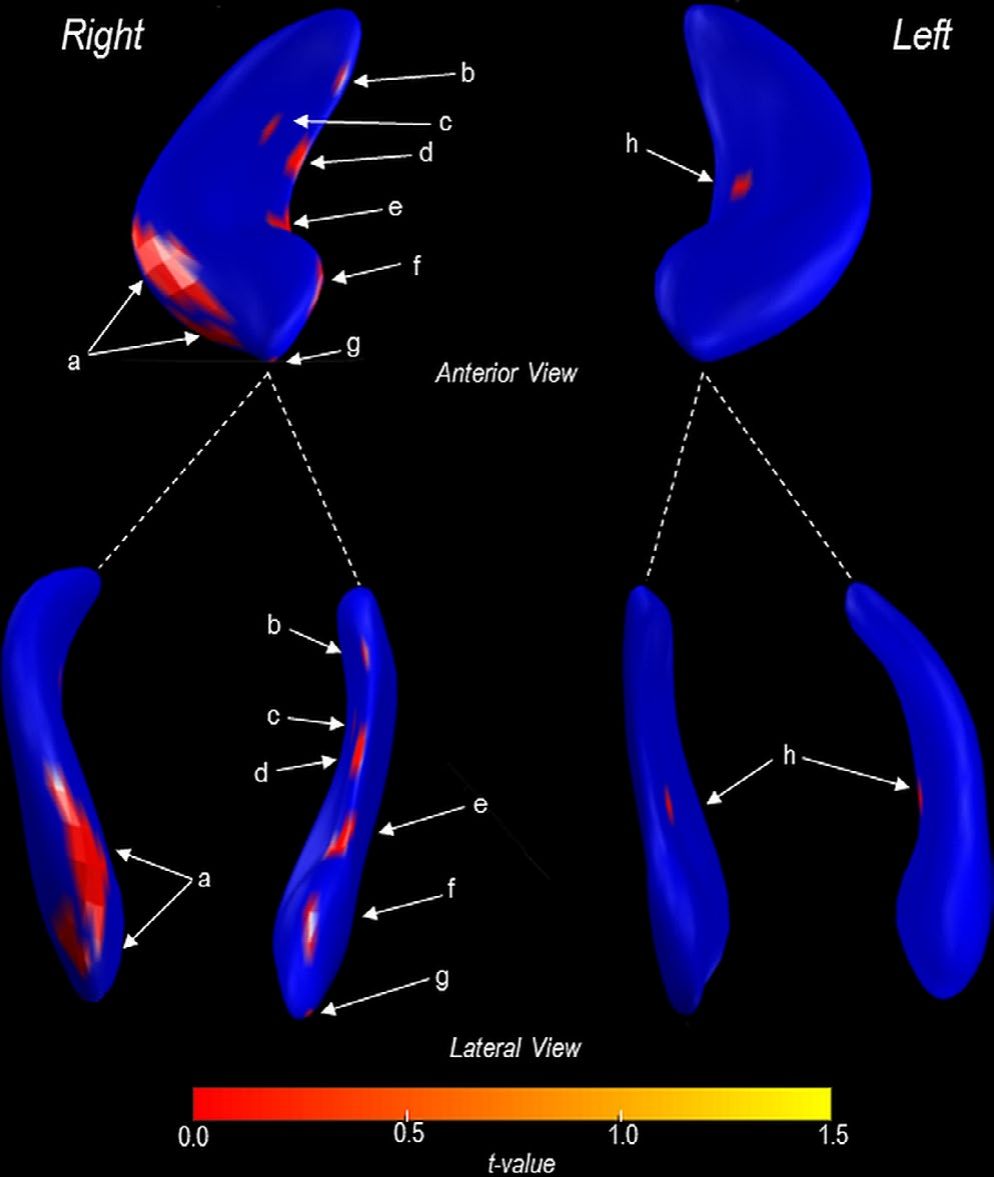
3D rendering of the left and right hippocampal volume changes in SVHD over healthy controls. Sites with localized changes were color‐coded and represented on the averaged 3D hippocampus surfaces across all subjects. The right hippocampus presents with a more decrease in volume over the left and included CA1 (a, g), the subiculum (b, d, e, f), CA4 (c), and dentate gyrus (h)

In addition, 3D surface morphometry shows significantly reduced volumes in the right compared with the left hippocampus in SVHD over control subjects (Figure 3). These sites included CA1 (Figure 3a,g), the subiculum (Figure 3b,d,e), CA4 (Figure 3c), and the dentate gyrus (Figure 3h). Hippocampal volume differences continues even after controlling for SES, in addition to age and TBV.

### 3.4 Correlations between hippocampal volumes and memory, mood, and anxiety scores

Significant positive correlations appeared between the left and right hippocampal volumes of SVHD and controls and memory as measured by the WRAML2 GMI, total MoCA, delayed memory recall scores, and other measures of the MoCA (attention vs. left hippocampal volume and language vs. right hippocampal volume) (Table 4). The left and right hippocampal volumes did not show significant correlations with BAI (anxiety) and PHQ‐9 (depression) scores (Table 4). Only clinical risk factor (e.g., ventricular type, number of surgeries, socioeconomic status, baseline oxygen saturation, and number of medications) associated with both left and right hippocampal volumes was baseline oxygen saturation (Left: *r *= 0.408, *p *= .007; Right: *r *= 0.385, *p *= .011). Furthermore, significant functional differences persist between groups, even controlling for SES.

**Table 4:** Partial correlations of SVHD and controls hippocampal volumes with memory, anxiety, and mood scores

Variables	Left hippocampal volume		Right hippocampal volume	
WRAML2 GMI	*r* = 0.32	*p* = .01*, *df* = 59	*r* = 0.28	*p* = .03*, *df* = 59
Verbal Memory Index (GMI)	*r* = 0.22	*p* = .10, *df* = 59	*r* = 0.19	*p* = .15, *df* = 59
Visual Memory Index (GMI)	*r* = 0.21	*p* = .10, *df* = 59	*r* = 0.19	*p* = .15, *df* = 59
Attention/Concentration (GMI)	*r* = 0.19	*p* = .14, *df* = 59	*r* = 0.20	*p* = .12, *df* = 59
WRAML2 GRI	*r *= 0.21 (*n* = 62)	*p* = .11, *df* = 58	*r *= 0.20 (*n* = 62)	*p* = .13, *df* = 58
Working Memory Index (GRI)	*r *= −0.05	*p* = .70, *df* = 59	*r* = 0.07	*p* = .57, *df* = 59
Verbal Recognition Index (GRI)	*r *= 0.16 (*n* = 62)	*p* = .21, *df* = 58	*r *= 0.22 (*n* = 62)	*p* = .09, *df* = 58
Visual Recognition Index (GRI)	*r* = 0.15	*p* = .24, *df* = 59	*r* = 0.22	*p* = .10, *df* = 59
MoCA Total	*r* = 0.33	*p* = .01*, *df* = 59	*r* = 0.32	*p* = .012*, *df* = 59
Visuospatial/Executive Function (MoCA)	*r* = 0.20	*p* = .12, *df* = 59	*r* = 0.16	*p* = .23, *df* = 59
Naming (MoCA)	*r* = 0.07	*p* = .60, *df* = 59	*r* = 0.009	*p* = .94, *df* = 59
Attention (MoCA)	*r* = 0.26	*p* = .04*, *df* = 59	*r* = 0.25	*p* ** = **.056, *df* = 59
Language (MoCA)	*r* = 0.24	*p* ** = **.06, *df* = 59	*r* = 0.26	*p* = .04*, *df* = 59
Abstraction (MoCA)	*r* = 0.21	*p* = .11, *df* = 59	*r* = 0.25	*p* = .05, *df* = 59
Delayed Memory Recall (MoCA)	*r* = 0.35	*p* = .006*, *df* = 59	*r* = 0.32	*p* = .01*, *df* = 59
Orientation (MoCA)	*r* = 0.12	*p* = .37, *df* = 59	*r* = 0.15	*p* = .25, *df* = 59
BAI	*r* = 0.02	*p* = .86, *df* = 59	*r *= −0.13	*p* = .32, *df* = 59
PHQ‐9	*r *= −0.18	*p* = .18, *df* = 59	*r *= −0.17	*p* = .19, *df* = 59

Abbreviations: age, and total brain tissue volume; BAI, Beck Anxiety Inventory; *df*, degrees of freedom; GRI, General Recognition Index; MoCA, Montreal Cognitive Assessment; PHQ‐9, Patient Health Questionnaire 9; WRAML‐2 GMI, Wide Range Assessment of Memory and Learning Version 2 General Memory Index. Covariates.

* *p* < .05. Signifying significance.

### 3.5 Structural brain MRI findings

Abnormal brain MRI findings in the SVHD group were 8 out of 25 (32%) compared with 2 out of 38 (5%) in the control group (Table 5). Cerebral lesions were detected in 5 (20%) with SVHD consisting of white matter changes, old infarctions/strokes, and periventricular volume loss. Incidental developmental abnormalities consisted of Rathke's cleft, pterygoid or perivascular region cysts in both groups. No correlations appeared in between participants with cerebral lesions and hippocampal volumes. Furthermore, after excluding 5 patients with overt cerebral lesions, the hippocampal volumes remained significant.

**Table 5:** Structural brain MRI findings in SVHD and controls

Variable	SVHD (*n* = 25)	Controls (*n* = 38)
Any abnormality *n* (%)	8 (32%)	2 (5%)
Focal or multifocal abnormality		
Focal infarction or atrophy	5 (20%)	0 (0%)
Developmental abnormality		
Minor*	3 (12%)	2 (5%)

* Minor malformations include Rathke's cleft cyst, pterygoid cyst, pituitary stalk thickening, and perivascular region cyst.

## 4 DISCUSSION

Adolescents with SVHD, who have undergone Fontan completion, showed significantly reduced right hippocampal volumes compared with age‐ and sex‐matched controls, and these changes were localized in the subiculum, CA1, CA4, and dentate gyrus sites. Both left and right hippocampal volumes showed significant associations with worse cognitive scores, including memory, but not with anxiety and depression. Other studies that examined hippocampal volumes showed significant bilateral differences between school‐age children with CHD and controls (Fontes et al., 2019; Latal et al., 2016; Munoz‐Lopez et al., 2017). However, our findings could reflect the evaluation of only the SVHD subgroup compared to studies with mixed types of CHD (Fontes et al., 2019; Latal et al., 2016). By focusing on this subset with cyanotic CHD and excluding other risk factors, such as premature birth (<37 weeks gestation), previous stroke, cardiac arrest, or the use of ECMO, the present study was able to control for additional factors that may have influenced hippocampal volume (age and TBV). Furthermore, it is unclear whether other studies utilized similar exclusion criteria, which may have increased the effects of these additional variables on hippocampal volume. Moreover, two previous study used automated regional segmentation tools rather than gold standard manual tracing to analyze hippocampal volume in CHD (Fontes et al., 2019; Latal et al., 2016). The automated processes have disadvantages in calculation of precise hippocampal volumes due to the absence of clear boundaries between the hippocampus and the amygdala making the segmentation of these structures challenging (Chupin et al., 2009).

Brain abnormalities were detected in 32% of adolescents with SVHD, which is consistent with other similar studies (Bellinger et al., 2015). Our hippocampal volume findings showed no associations with a previous brain abnormality or cerebral infarction, which is similar to another study examining hippocampal volumes in the adolescent CHD population (Fontes et al., 2019). However, one study reported associations between brain abnormalities and smaller hippocampal volumes (Latal et al., 2016). These inconsistencies may result from inclusion criteria or severity classification of brain abnormalities.

Few clinical variables have made a contribution to explain smaller hippocampal volumes in studies with CHD or SVHD cohort (Fontes et al., 2019; Latal et al., 2016; Munoz‐Lopez et al., 2017). The only clinical factor in this study associated with hippocampal volumes was baseline oxygen saturation level and was positive with higher oxygen saturations associated with larger hippocampal volumes. Another study identified only hypothermic circulatory arrest to be associated with smaller hippocampal volumes (Fontes et al., 2019). The hippocampus is known to be sensitive to hypoxic changes, which is reflected in our findings and the other intraoperative variable.

One possible reason to explain the lateralization of hippocampal volume reduction in SVHD could be handedness. Most adolescents with SVHD in the study were right‐handed. As handedness is controlled by the contralateral brain hemisphere, the left brain may receive more blood, maintaining left hippocampal volume (Gur et al., 1982). However, similar proportion of control subjects were right‐handed, and left and right hippocampal volumes were comparable in control subjects, indicating that this possibility is less likely. Another potential explanation is that the majority of SVHD participants had a right modified Blalock–Taussig shunt (mBTS) for their first stage surgical palliation, which could create a “steal effect” during systole causing reduced cerebral blood flow through the right carotid artery. However, this has only been reported in a case study using right and left cerebral near‐infrared spectroscopy (NIRS), not intracranial Doppler, with reduced values seen in the right cerebral hemisphere compared with the left (Algra et al., 2011). Thus, compromised cerebral blood flow to the right hemisphere could result in hippocampal atrophy.

The SVHD patients were found to have reduced hippocampal volumes, which correlated to impairments in various aspects of memory including working memory, visual, and verbal memory, long‐term memory, short‐term memory, and delayed recall, with combined hippocampal volumes of SVHD and control subjects. The relationships between hippocampal volume and various memory abilities seen were consistent with other studies which determined hippocampal volume in different types of CHD (cyanotic and acyanotic) (Latal et al., 2016; Munoz‐Lopez et al., 2017). Lateralization in hippocampal brain volumes and association with memory has also been identified in other studies which demonstrated a rightward bias in short‐term and spatial memory storage/retrieval processes (Klur et al., 2009; Piekema et al., 2006) providing evidence that a decreased right hippocampal volume can affect specific memory tasks.

Population‐based studies in children with critical CHD have identified disparities associated with SES, Hispanic ethnicity, and worse health outcomes (Peyvandi et al., 2018). Our SVHD group were 50% Hispanic ethnicity and had lower SES compared with controls. After controlling for SES, there were still significant differences in hippocampal volumes and functional outcomes in the SVHD group compared with controls, and SES did not contribute significantly to those variables. However, other important factors of SES, such as maternal age and education may provide more of an impact than annual salary, as measured in this study.

Localized tissue changes in specific subfields of the hippocampus are still emerging in various disease specific populations. Our study identified atrophy predominantly in CA1, CA4, dentate gyrus, and the subiculum areas with some areas consistent in another CHD study (Fontes et al., 2019). The CA4 and dentate gyrus sites are involved in encoding, learning, and recall over shorter intervals while CA1 and the subiculum with retrieval functions and immediate/delayed visual and verbal memory (Zammit et al., 2017), which correlates with our memory findings.

While the present study determined positive correlations between memory and hippocampal volumes, anxiety or mood scores did not show such correlations. No prior research in CHD has linked hippocampal volumes and mood. While our study focused on gray matter differences, it is hypothesized that white matter regions or other areas of mood circuitry may play a significant role in mood outcomes, since other limbic areas are classically‐associated with mood and anxiety (i.e., amygdala, mammillary bodies, anterior thalamus, anterior cingulate, and insula). Studies have shown that lower cerebral blood flow to the limbic system is associated with anxiety and depression (Drevets et al., 2008), and decreased white matter was seen with hippocampal atrophy (Martin et al., 2009). In patients with SVHD, decreased cerebral blood flow and white matter may lead to worse anxiety and mood outcomes (Jimenez et al., 2018; Tanti & Belzung, 2013). Future research focusing on fiber tractography to study connections between the hippocampus and other limbic structures is needed to help determine whether anxiety and mood impairments are localized to white matter in individuals with SVHD. An alternative explanation is that anxiety and mood functions may be localized to specific region of the hippocampus that may have been preserved in SVHD. Other studies have found that the ventral subregion of the hippocampus is more associated with anxiety and depression, while learning and memory are thought to be localized to the dorsal hippocampus (Kaltman et al., 2005; Licht et al., 2004). Though anxiety and depression were a significant finding between groups, the scores identified mild impairment in the SVHD group, which could also explain the lack of associations with hippocampal volumes.

Although the mechanism of hippocampal pathology in SVHD is unclear but may include several processes. Studies have found that CHD causes alterations in cerebral blood flow, as well as decreased oxygenation, prenatally and after birth (Kaltman et al., 2005; Licht et al., 2004).

The hippocampal tissue has a high metabolic rate and requires a higher oxygen delivery during developmental periods, which is critical for growth and expansion of hippocampal neurons and their processes. Thus, it is likely to be highly vulnerable to any changes in cerebral perfusion (Cooper et al., 2015). SVHD is also associated with the risks of cardiopulmonary bypass surgery during infancy, which include the incidence of cerebral macroemboli, microemboli, and periods of brain hypoxia and ischemia. Hypoxic conditions have been previously implicated in hippocampal damage and memory impairment (Munoz‐Lopez et al., 2017). Additionally, postsurgery, some patients may develop ischemic strokes or cerebral venous thromboses that can affect hippocampal tissue (Latal et al., 2016) Subsequent operations and prolonged hospital stays may contribute to increased risk of these postsurgery complications. Finally, other process, such as hypotension and an irregular cerebrovascular autoregulation mechanism, may be factors in hippocampal volume reduction in SVHD.

At this time, there are no prenatal treatments or prevention for SVHD, and surgery is the most common treatment option. Since hippocampal volume reductions correlate with memory impairment in adolescence, treatment is needed at an early age and should focus on increasing neurogenesis, neuroprotection, and improving memory and cognitive outcomes. However, methods to foster neurogenesis in SVHD patients have not yet been determined. In other types of brain injury (i.e., strokes and traumatic brain injury), medications (statins) (Lu et al., 2007), exercise (Liu et al., 2009), and cognitive exercises (Maguire et al., 1997) have been used to promote neuroplasticity. In other studies, early parental care (i.e., affection, verbal responsivity) correlated with hippocampal volume (Farah et al., 2008) and memory development (Rao et al., 2010). Despite recent trends in standardized early intervention and neurodevelopmental follow‐up postsurgical palliation, the benefits of these programs in terms of long‐term neurocognitive outcomes still remain unclear.

### 4.1 Limitations

Despite the small sample size, significant differences in hippocampal volumes emerged between groups. The lateralization finding may also be due to multiple statistical tests performed. However, a Bonferroni correction identified that *p*‐values ≤ .001 remained statistically significant, despite the small sample, but due to the large effect size. While the extensive exclusion criteria made a more homogeneous SVHD sample (gestation < 37 weeks, no previous stroke, ECMO use, cardiac arrest, pacemaker), this may reflect a group with better health within their chronic condition, and thus, may not be generalizable to all SVHD participants. Secondary to a small sample size, we combined SVHD and control participants to examine correlations, and further studies are required to assess such correlations in SVHD participants only. Intelligence quotient (IQ) was not measured as part of this study and were unable to discern if the memory impairment is isolated or part of general IQ reduction. However, we were able to perform a more extensive memory assessment than provided in an IQ test. In addition, there is the potential for subjective bias related to manual ROI tracings compared with automated programs. However, manual tracing is still considered the gold standard. Our tracer was blinded to group with excellent intra‐ and inter‐rater reliabilities. Lastly, without serial brain MRIs, we are unable to specify the timing or mechanism of injury leading to smaller hippocampal volumes due to the cross‐sectional study design.

## 5 CONCLUSIONS

In adolescent with SVHD who have undergone surgical palliation, the hippocampus shows reduced volumes, which are localized in CA1, CA4, dentate gyrus, and the subiculum sites and correlate with worse memory performance. The hippocampal volume changes provide evidence that the memory deficits, commonly reported in SVHD, have a brain structural etiology and are not solely due to adolescent behavior or family dysfunction. These findings indicate the need to identify potential interventions to improve memory and mood deficits and access longitudinal progression of memory, mood, and hippocampal brain changes in this aging population.

## CONFLICT OF INTEREST

All authors have no conflicts of interest to declare at the time of submission.

## AUTHOR CONTRIBUTIONS

NAP, BR, RK, and MAW: contributed to concept and design, methodologic expertise, MRI data acquisition and statistical analysis, interpretation of results, drafting and revising manuscript, and final approval. NJH and ABL: contributed to participant recruitment, content expertise, editing critical clinical aspects of the manuscript, and final approval. SM and CCM: contributed to the methods of this manuscript and provided hippocampal volume tracings, inter‐rater reliability, figure development, writing sections of the manuscript, and final approval.

### Peer Review

The peer review history for this article is available at https://publons.com/publon/10.1002/brb3.1977.

## Data Availability

The data that support the findings of this study are available from the corresponding author upon reasonable request.
